# How environmental regulation affects China’s rare earth export?

**DOI:** 10.1371/journal.pone.0250407

**Published:** 2021-04-22

**Authors:** An Pan, Shuangshuang Feng, Xinyuan Hu, Yaya Li

**Affiliations:** 1 School of Economics, Zhongnan University of Economics and Law, Wuhan, Hubei, China; 2 School of Finance & Economics, Jiangsu University, Zhenjiang, Jiangsu, China; Institute for Advanced Sustainability Studies, GERMANY

## Abstract

China’s rare earth export trade has developed so rapidly since 1990s that China has gradually occupied a leading position in the international market. However, this fast development was proceeding at the cost of the rare earth energy consumption and environmental devastation. Now China begins to attach great importance to environmental protection, which attracts many researchers. This study aims to analyze the influence of environmental regulation on China’s rare earth export trade. And the original study is amongst the few to examine the relationship between environmental regulation and China’s rare earth export with the product-level data. Different from previous studies, this paper selects China’s rare earth export data from 1995 to 2015 and introduces product heterogeneity based on the rare earth production process. Moreover, this study uses the entropy weight method to measure the intensity of environmental regulation. The core conclusions are as follows: (1) Environmental regulation significantly promotes rather than restrains China’s rare earth export. (2) According to the rare earth production process, this paper divides rare earth products into 3 kinds, that is, rare earth raw materials, rare earth useful components and rare earth end-use applications. Then, it is found that rare earth useful component export in processing and smelting is positively affected by environmental regulation. Rare earth raw materials and end-use applications in China’s export are hardly affected. (3) Technological innovation has a mediating effect on the impact mechanism of environmental regulation on China’s rare earth export, which means that environmental regulation significantly promotes technological innovation of enterprises, and thereby the rare earth export is increased. The findings are helpful for policymakers to resolve the issue of environmental devastation.

## Introduction

Rare earth (RE) is widely used in modern industry such as new energy, new materials, energy conservation, aeronautics and astronautics, and electronic information on account of its special physical properties [1]. RE is also listed as a kind of critical materials by U.S. Department of Energy [2]. Meanwhile, Chinese RE industry has grown quickly since 1990s and Chinese RE mining and refining technology has been upgraded all the time. In 2000, China became the largest RE producer and exporter in the world. At present, China takes a lead in the RE international market because China supplies more than four fifths of global RE demand with its 37 percentage of world reserves according to the report by U.S. Geological Survey (USGS).

However, a huge price has been paid behind industrial progress, that is, resource overconsumption and environmental pollution. On the one hand, China’s RE reserves have fallen dramatically in recent years as a result of resource overexploitation, suggesting that China’s mineral wealth is drying up. According to USGS, China’s RE reserves once accounted for more than 60% of the world’s total reserves, but that proportion fell to 37% in 2018. In addition, Situation and Policies of China’s Rare Earth Industry published in 2012 recorded that only a third of the original volume of RE resources in Baotou’s main mining areas were available, and the reserve-extraction ratio of ion-absorption RE mines in China’s southern provinces had descended from 50 to 15 between 1990s and 2010s. Over the past 30 years, the growth of RE industry in Baotou has damaged the environment seriously [3]. On the other hand, environmental contamination caused by RE mining and refining needs to be resolved urgently as well. New York Times reported that the excessive exploitation of RE metals and radioactive leaks in the process of RE refining had brought serious ecological environmental pollution in Tianjin, Jiangxi province and Guangdong province in China [4]. With an emphasis on growing environmental awareness, the utilization strategy needs to be adopted to ensure sustainable growth in the prevailing competitive and challenging international situation [5, 6]. Therefore, Chinese government promulgated a series of RE export restrictions covering implementing RE product export duties, export quotas and the administration and allocation of the export quotas in order to achieve the sustainable development of RE industry [7]. Then the United States, the European Union and Japan strongly opposed these measures and litigated to WTO in 2009. Ministry of Ecology and Environment of the People’s Republic of China enacted Emission Standards of Pollutants in Rare Earth Industry to limit the discharge value of pollutants in RE companies in 2011. Unfortunately, it has failed to restrict China’s RE export and even accelerates the depletion of RE industry [8]. Afterwards, China has been attaching importance to environmental protection and enhancing environmental regulation to control pollution problems caused by RE dressing and smelting. Therefore, this study aims to find out the association between environmental regulation and China’s RE export, and explore the mechanism by which environmental regulation affects RE export.

Two main points of view emerged from the preliminary studies of the influence of environmental regulation on the export. The conventional view holds that environmental regulation is detrimental to the export because it imposes additional costs on enterprises and could undermine their competitiveness, and therefore has a negative impact on the export [9–12]. First, the internal costs of existing enterprises will be affected to some extent by environmental regulation, so that product costs and prices rise. Existing enterprises tended to buy lots of pollution abatement equipment and improve production technologies simultaneously for meeting pollutant discharge standards [13]. Du et al. found that severe environmental pollution had made RE export prices lower in China and was detrimental to the development of environmental sustainability [14]. After implementing regulations, the price of China’s RE products has been increasing for an extended period of time [15]. Next, some studies examined that environmental regulation brought external costs to existing enterprises, which could be invoked as the reason why there was a negative link between environmental regulation intensity and export [11, 16–18]. Thus, this had received considerable attention in the research area, and the Pollution Heaven Hypothesis emerged. This hypothesis assumes that changes in environmental regulation have led to a shift in pollutant production from countries with strict environmental regulation to countries with lax environmental regulation [17]. Supported by evidence based on gravity models in 21 OECD countries, Van Beers and Van Den Bergh pointed out that strong environmental regulation has resulted in the loss of competitiveness in pollution intensive industries, as well as reduced export and increased import [18].

By contrast, the Porter Hypothesis (PH) declares that well-designed environmental regulation can enhance existing firms’ global competitiveness. Over the past 30 years, numerous economists had found conflicting evidence and alternative theories to explain the PH, proving that environmental regulation such as taxes or emission allowances can trigger innovation that may offset the costs of firms, which can be called “innovation offsets” effect in some circumstances [19–21]. Then, Jaffe and Palmer broadened the PH to include three forms: “weak”, “strong” and “narrow” [22]. At the beginning, “weak” version of the PH finds that environmental regulation could stimulate technology innovation of existing firms [23, 24]. In quantitative terms, Yang et al. used industry-level panel data to examine whether environmental regulation was related with R&D and productivity in Taiwan, revealing that stronger environmental protection of manufacturing industry induced more R&D and enhanced industrial competitiveness [25]. Rubashkina et al. found that there was a positive relationship between environmental regulation and the output of innovation activity in European manufacturing sectors [26]. And, Li et al. found that both environmental regulation and financial development played a role in promoting green technology [27]. Secondly, “narrow” version of the PH believes that only appropriate environmental regulation could promote technological innovation of existing firms. A specific niche noticed that market-based instruments (MBI) for environmental regulation might over time have obviously greater, positive influence than command-and-control (CAC) approaches on the invention, innovation, and popularization of ideal environmental protection technology [28, 29]. He et al. found that Chinese RE export policies currently result in accelerating its depletion and used a multi-attribute decision-making method to select the optional trading partner for China [8]. Finally, “strong” version of the PH explains the correlation between environmental regulation and the export, increasing the export of existing firms by stimulating innovation [30, 31]. Costantini and Mazzanti explored that the export competitiveness of European Union was fostered by environmental regulation and innovation, testing the “strong” version of the PH [32]. While, Stavropoulos et al. concluded the U-shaped relationship between environmental regulation and industrial competitiveness [33]. Hence, environmental regulation is conducive to expand the export by enlarging industrial competitiveness [34–36].

Although innovation helps firms to achieve valuable benefits [37, 38], technological innovation has resulted in some negative changes in the society [39]. More attention is paid to RE industry in this study. RE is an important strategic mineral resource, but its production causes serious environmental pollution, especially during RE processing and smelting [40]. As the largest RE producer in the world, China is particularly susceptible to environmental destruction such as water pollution, air pollution and soil erosion with the upgrade of technological innovation [40, 41]. Chen et al. showed that the exploitation of RE elements had caused serious soil erosion and soil pollution in China [42]. Thus, firms need to take the environmental responsibility while making more profit through technological innovation. Moreover, environmental regulation has become a vital measure for China’s government to take in order to achieve the sustainable development of RE industry, and it is tested that different environmental regulations have distinct effects on different RE products [43, 44]. Wang et al. found that China had gradually tended to reduce the RE production and export and showed that the RE international network was unstable [45].

A gravity model is used to test the impact of environmental regulation on the export in this research. This study aims to identify and explain the relationship between environmental regulation and China’s RE export, and verify that technological innovation has a mediation effect on the mechanism. To our best knowledge, this study is the first one to use RE product-level data to explore how environmental regulation affects China’s RE export and analyze product heterogeneity in the process. Moreover, this study uses the entropy weight method (EWM) to measure the intensity of environmental regulation, increasing the robustness of the results.

The frame of the paper is organized as follows. It starts in Section 1 with introduction and literature review, explaining the theoretical framework of this paper. Next, Section 2 measures the intensity of environmental regulation, designs the model and introduces the data. Then, the findings analyze the empirical results in Section 3. Section 4 enters the realm of discussion. Finally, it is ended with Section 5, outlining the major research gaps that remain to be filled in this important area, as well as policy recommendations for China.

## Materials and methods

### Model

#### Benchmark model

Based on the theoretical framework in Section 1, we construct an extended gravity model according to Anderson and van Wincoop [46], taking the intensity of environmental regulation into consideration. The following basis model tends to investigate the impact of environmental regulation on the export:

$$lnEXP_{ijt}=\alpha_{0}+\alpha_{1}ER_{it}+\alpha_{2}Control_{ijt}+\varepsilon_{ijt}$$ (1)

where *ln* represents the natural logarithm applied to a variable to produce more stationary data; *i*, *j* and *t* represent China, trade partners of China and year; *EXP*_*ijt*_ denotes China’s RE export to country *j* in year *t*; *ER*_*it*_ represents China’s environmental regulation intensity in RE industry at time *t*; control variables include gross domestic product (GDP) of China in year *t* (*lnGDP*_*it*_), GDP of trade partners at time *t* (*lnGDP*_*jt*_), the geographic distance between China and country *j* (*lnDis*_*ij*_) denominated in kilometers, absolute value of the difference in per capita GDP of China and country *j* in year *t* (*lnpgdp*_*ijt*_), economic freedom index of country *j* at time *t* (*IEF*_*jt*_), dummy variable stating whether the trade partner has a RE dispute with China in year *t* (*TF*_*ijt*_) and total factor productivity (TFP) of country *j* at time *t* (*TFP*_*jt*_); and *ε*_*ijt*_ is the random error term.

#### Mediation effect model

In order to further explore the effect mechanism of environmental regulation on the export. We refer to the mediation effect test proposed by Baron and Kenny [47], involving three steps shown in Eqs (1)–(3):

$$TFP_{it}=\beta_{0}+\beta_{1}ER_{it}+\beta_{2}Control_{ijt}+\varepsilon_{ijt}$$ (2)

$$lnEXP_{ijt}=\gamma_{0}+\gamma_{1}ER_{it}+\gamma_{2}TFP_{it}+\gamma_{3}Control_{ijt}+\varepsilon_{ijt}$$ (3)

where *TFP*_*it*_ represents TFP of China in year *t*. The coefficient (*α*_*1*_) of Eq (1) is the total effect of environmental regulation on China’s RE export; the coefficient (*β*_*1*_) of Eq (2) is the effect of environmental regulation stringency on the mediator variable (*TFP*_*it*_); the coefficient (*γ*_*2*_) of Eq (3) is the effect of the mediator variable (*TFP*_*it*_) on the export after controlling the possible influences; the coefficient (*γ*_*1*_) is the direct effect of environmental protection on China’s RE export after controlling the effect of the mediator variable (*TFP*_*it*_). Eq (3) can be tested under the condition that the regression coefficient estimates of *ER*_*it*_ in Eqs (1) and (2) are significant. In Eq (3), if the regression coefficient estimate of the core explanatory variable (*ER*_*it*_) is not significant, but the regression coefficient estimate of the mediator variable (*TFP*_*it*_) is significant, indicating that *TFP*_*it*_ has a complete mediation effect. If both coefficient estimates are significant, but *γ*_*1*_ is smaller than *α*_*1*_, then *TFP*_*it*_ has a partial mediation effect.

### Variables

#### Dependent variable

**Fig 1:**
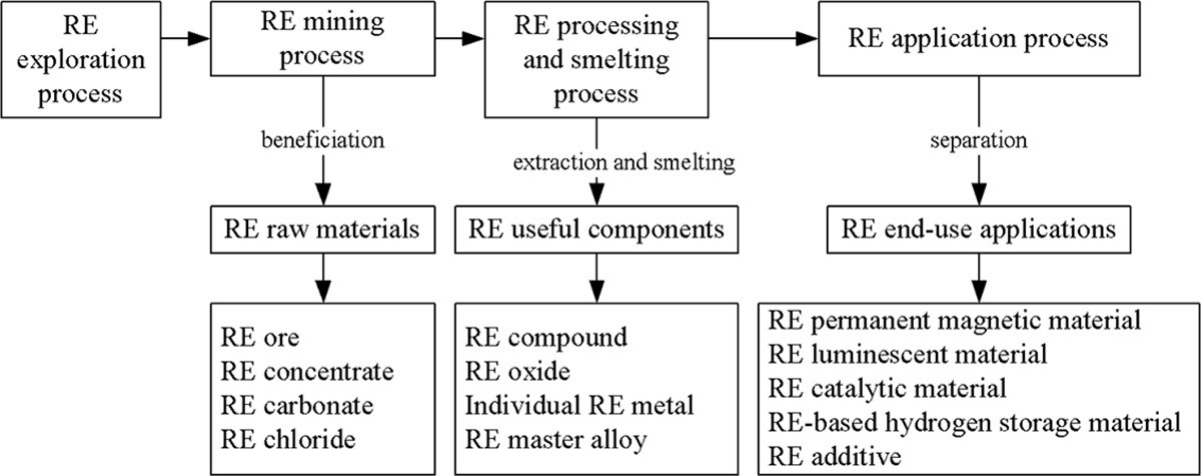
The production process of RE products.

The dependent variable in the econometric model shown in Eq (1) is the export volume of RE industry in China. In the analysis of product heterogeneity, RE products are divided into RE raw materials, RE useful components and RE end-use applications [48]. They are produced in three processes, that is, the primary production, RE processing and smelting and the final production used for functional products respectively according to the production process. What’s more, Fig 1 shows the production process of RE products. Moreover, their HS codes are listed in Table 1 and the export volumes of the three kinds of products are the dependent variables in the sub-sample analysis. In the robustness of product heterogeneity, the exports of cerium compounds, compounds of rare earth metals excluding cerium, ferro-alloys and magnets are the dependent variables.

**Table 1:** HS codes of RE products.

Type	HS code	Product
RE raw materials	253090	Mineral substances
RE useful components	280519	Alkali or alkali-earth metals, other than sodium and calcium
	280530	Rare earth metals
	284610	Cerium compounds
	284690	Compounds of rare earth metals excluding cerium
	360690	Ferro-cerium and other pyrophoric alloys in all forms
RE end-use applications	320650	Inorganic products of a kind used as luminophores
	720299	Ferro-alloys
	720521	Alloy steel powders
	850511	Magnets

#### Explanatory variable

**Fig 2:**
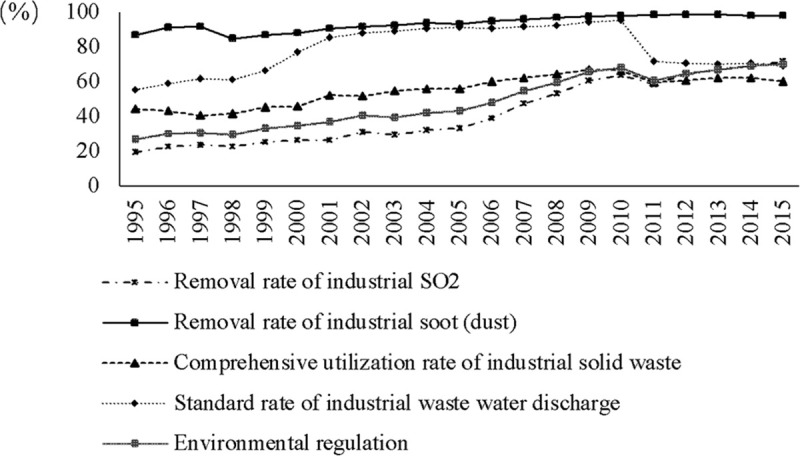
Calculation results of environmental regulation.

The core explanatory variable in the basic model is environmental regulation (*ER*_*it*_) calculated as follows and reported in Fig 2.

In this paper, the variable of environmental regulation (*ER*_*it*_) is constructed to measure the intensity of implementing environmental policies in China’s RE industry. Accordingly, 4 indicators are selected to measure environmental regulation, including: (1) Removal rate of industrial SO_2_, (2) Removal rate of industrial soot (dust), (3) Comprehensive utilization rate of industrial solid waste, (4) Standard rate of industrial waste water discharge [49]. The calculation process of 4 indicators is shown in Table 2.

**Table 2:** Comprehensive indexes of environmental regulation.

Aim	Indicators	Calculation
Environmental regulation comprehensive indexes	Removal rate of industrial SO_2_	Industrial SO_2_ removal / Industrial SO_2_ emission
	Removal rate of industrial soot (dust)	Industrial soot (dust) removal / Industrial soot (dust) emission
	Comprehensive utilization rate of industrial solid waste	Disposal volume of industrial solid waste / Generation volume of industrial solid waste
	Standard rate of industrial waste water discharge	Standard-meeting discharge of industrial waste water / Discharge of industrial waste water

Then we adopt a comprehensive index method to construct *ER*_*it*_, using the EWM to determine the weights of the above indexes in Table 2, which is different from the previous researches [50–52]. The EWM was developed by Shannon [53] and proposed as a measurement for uncertainty in information and formulated in terms of probability theory. In a matrix of *h* × *t* values, *h* (1 ≤ *h* ≤ m) represents the total number of different indicators and *t* (1 ≤ *t* ≤ n) means the total number of years, and the next specific steps are as follows [54].

First, each individual indicator of pollutant discharge is standardized as follows. Each value of the matrix is normalized to the range of [0,1]. The normalization distinguishes between positive and negative indicators, as shown in Eq (4).

$$x^{*}{}_{ht}=\{{}_{\frac{max(x_{ht})‐x_{ht}}{max(x_{ht})‐min(x_{ht})},\;if\;h\;is\;a\;negative\;indicator}^{\frac{x_{ht}‐min(x_{ht})}{max(x_{ht})‐min(x_{ht})},\;if\;h\;is\;a\;positive\;indicator}$$ (4)

All pollutant discharge indicators in the study are positive, so the first equation in Eq (4) is selected to normalize data. *x*_*ht*_ denotes the original value of the indicator *h* in year *t*. And, *max(x*_*ht*_*)* and *min(x*_*ht*_*)* are the maximum and minimum values of the indicator *h* among all years respectively, and x^*^_ht_ is the standardized value of the indicator *h*.

Second, entropy quantification is as follows. This step aims to quantify the information of each indicator, requiring a probability parameter. Ordinarily, this probability parameter in the calculation of Shannon entropy is determined by the frequency of occurrence of different values [55].

$$\{{}_{e_{h}=\frac{1}{ln(n)}\sum_{t=1}^{n}p_{ht}ln(p_{ht})}^{p_{ht}=\frac{x^{*}{}_{ht}}{\sum_{t=1}^{n}x^{*}{}_{ht}}}$$ (5)

In the equation, the probability parameter is defined as the proportion (*p*_*ht*_) of the annual value of an indicator to the sum of the values of this indicator for all years, and *e*_*h*_ is the entropy quantification of the indicator *h*.

Third, the equation for determining the weight (*w*_*h*_) of the indicator *h* is expressed as follows.

$$w_{h}=\frac{1‐e_{h}}{\sum_{h=1}^{m}e_{h}}$$ (6)

Finally, environmental regulation comprehensive index is shown in Eq (7), which is the core explanatory variable in the empirical analysis, and the calculation results of environmental regulation are put in Fig 2.

$$ER_{t}=\sum_{h=1}^{m}w_{h}p_{ht}$$ (7)

#### Control variables

Following Van Beers and Van den Bergh [18], Cantore and Cheng [21] and Abbasi et al. [56], there are seven control variables being added into the econometric model in this paper: GDP (*GDP*_*it*_ and *GDP*_*jt*_), the geographic distance (*Dis*_*ij*_), absolute value of the difference in per capita GDP (*pgdp*_*ijt*_), economic freedom index (*IEF*_*jt*_), dummy variable stating whether the trade partner has a RE dispute with China (*TF*_*ijt*_) and total factor productivity (*TFP*_*jt*_). *GDP*_*it*_ and *GDP*_*jt*_ in this paper represent supply capacity of China and spending power of the importer, respectively. *Dis*_*ij*_ measures the capital’s distance and reflects the level of transport cost between two countries. Based on the Linder hypothesis [57], the smaller *pgdp*_*ijt*_ is, the larger scale of trade in two countries will be. *IEF*_*jt*_ consists of trade, currency, finance and investment, showing the economic openness of the trade partner. *TF*_*ijt*_ aims at controlling the influence of RE dispute on the export in China. *TFP*_*jt*_ is the representative variable of technological innovation.

#### Mediation variable

The PH shows that appropriate environmental regulation can promote the export through enhancing technological innovation. As the representative variable of technological innovation, China’s TFP (*TFP*_*it*_) is selected as the mediation variable.

### Data sources

In view of data availability and validity, the final panel sample covers 17 countries from 1995 to 2015. These countries are main importers of China’s RE products, including Australia, Belgium, Brazil, Canada, England, France, Germany, India, Italy, Japan, Korea, Mexico, Netherlands, Russia, Spanish, Turkey and USA. Table 3 shows the statistical descriptions of these variables.

**Table 3:** Statistical descriptions.

Symbol	Index	Mean value	Standard error	Minimum value	Maximum value
ln*EXP_g*	Export of RE products	15.745	2.421	1.099	20.097
ln*EXP_1g*	Export of RE raw materials	13.521	4.455	0.000	20.009
ln*EXP_2g*	Export of RE useful components	12.012	3.640	0.000	17.186
ln*EXP_3g*	Export of RE end-use applications	14.717	2.584	0.000	17.872
*ERi*	Environmental regulation	48.397	15.106	26.911	70.350
ln*GDPi*	China’s GDP	28.627	0.898	27.323	30.030
ln*GDPj*	Trade partner’s GDP	27.857	0.965	25.856	30.534
ln*Dis*	Geographic distance	8.830	0.668	6.862	9.738
ln*pgdp*	Absolute value of the difference in per capita GDP	9.610	1.178	5.463	11.030
*IEF*	Economic freedom index	66.796	8.899	45.100	83.100
*TF*	Whether related to the RE case	0.076	0.265	0.000	1.000
*TFPj*	Trade partner’s TFP	0.807	0.219	0.266	1.198
*TFPi*	China’s TFP	0.369	0.056	0.286	0.434

**Fig 3:**
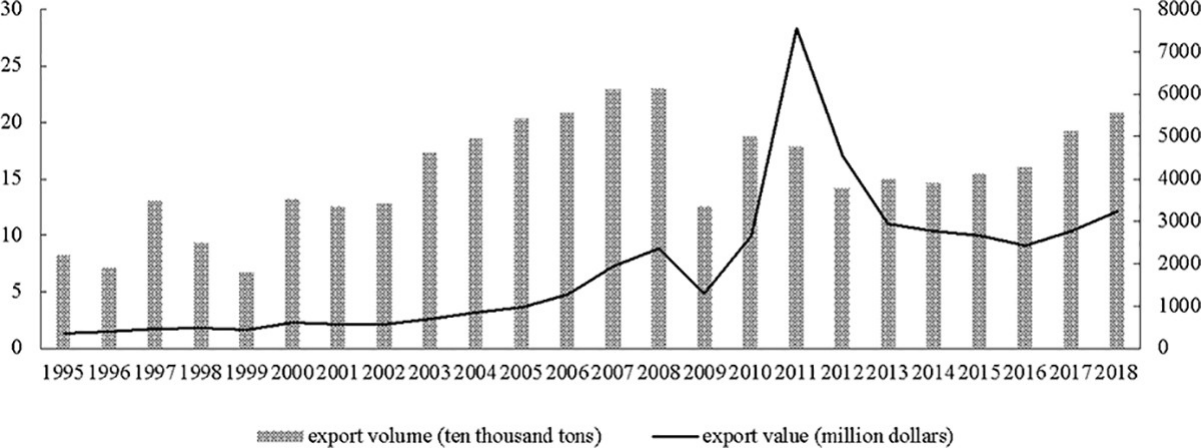
Total export volume and value of China’s RE products from 1995 to 2018. The main axis of the vertical axis represents the export volume.

**Fig 4:**
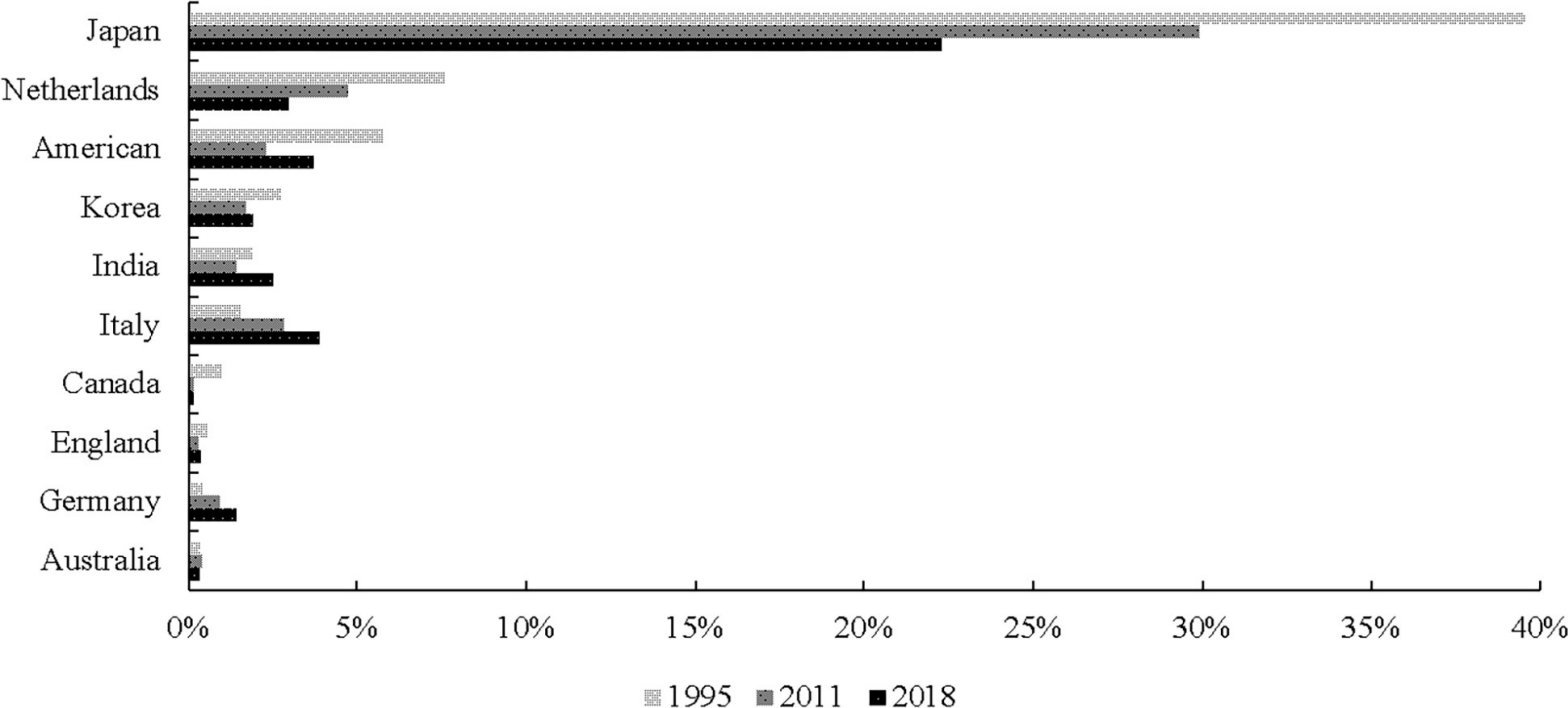
The top ten importers of China’s RE products.

The export volume data of RE industry in China are derived from UN Comtrade Database. Total export volume and value of China’s RE products from 1995 to 2018 are shown in Fig 3. It shows that export volume decreased and export value increased since 2010 due to taking environmental regulation into effect. In addition, Fig 4 shows the top ten importers of China’s RE products, reflecting that Japan is the largest importer. Environmental regulation data are retrieved from China Statistical Yearbook on Environment and China City Statistical Yearbook. GDP data are obtained from World Development Indicators. And geographic distance variables are from CEPII Database. Furthermore, the economic freedom index is derived from the Heritage Foundation’s annual report. In addition, countries related to the RE case are shown on European Union website. TFP data are derived from Penn World Table 9.1.

## Results

### Benchmark regression

The generalized least square (GLS) is adopted to conduct benchmark regression [58], and the results are shown in Table 4. There are two reasons for using GLS: the one is that GLS helps to cope with the problems of serial correlation and heteroscedasticity [59], and the other is that the fixed effect is not available because the variable of geographical distance is unchangeable and unable to be estimated [60].

**Table 4:** Benchmark regression results.

Variable	Model (1)	Model (2)	Model (3)	Model (4)	Model (5)	Model (6)
*ERi*	0.056***	0.019***	0.017***	0.017***	0.021***	0.024**
	(23.68)	(4.06)	(2.90)	(2.99)	(2.61)	(2.22)
ln*GDPi*		0.223***	0.252**	0.247**	0.172	0.236
		(2.82)	(2.55)	(2.57)	(1.25)	(1.23)
ln*GDPj*		1.049***	1.053***	1.002***	1.000***	1.096***
		(79.16)	(76.55)	(65.43)	(66.54)	(73.84)
ln*Dis*			-1.098***	-1.105***	-1.105***	-1.272***
			(-93.15)	(-90.22)	(-73.61)	(-66.62)
ln*pgdp*				0.089***	0.041**	-0.326***
				(6.62)	(2.33)	(-14.56)
*IEF*					0.009***	0.017***
					(6.32)	(8.19)
*TF*					-0.072	-0.118
					(-1.21)	(-1.55)
*TFPj*						2.029***
						(29.67)
Constant	13.029***	-20.754***	-11.922***	-11.149***	-9.306***	-11.079**
	(108.36)	(-10.04)	(-4.62)	(-4.44)	(-2.58)	(-2.20)
Observations	357	357	357	357	357	340

Notes: Robust t-statistics are in parentheses

*, **, and *** refer to the significance levels of 10%, 5%, and 1%, respectively.

Model (1) has no control variables, only considering the effect of environmental regulation on China’s RE export. The estimated coefficient of the core explanatory variable, the intensity of domestic environmental regulation (*ERi*), is 0.056 at the significance level of 1%, proving that environmental regulation can significantly promote the export of China’s RE products instead of restraining. The stronger the domestic environmental regulation is, the larger the export volume of China’s RE products will be, proving that the PH is true. Moreover, control variables in Eq (1) involving GDP of two countries (ln*GDPi* and ln*GDPj*), geographic distance between two countries (ln*Dis*), absolute value of the difference in per capita GDP of two countries (ln*pgdp*), economic freedom index of trading partners (*IEF*), dummy variable stating whether the trade partner has a RE dispute with China (*TF*) and TFP of trade partners (*TFP*) are added into Model (2)-(6) in sequence. What can be found is that the estimated coefficients of environmental regulation indicator in Model (2)-(6) are significantly positive, which reveals that environmental regulation has a steady positive effect on China’s RE export.

In particular, this study mainly pays attention to the results of Model (6), where the estimated coefficient of environmental regulation is 0.024, which is positive at the significance level of 5%. To make it specific, 0.024 means that China’s RE product export rises 2.4% with 1% increase in environmental regulation stringency. The above theoretical framework can help to explain the following results: with the enforcement of environmental protection policies and the enhancement of environmental protection consciousness, China’s RE enterprises improve their product competitiveness through technological innovation, thus promoting China’s RE export, which is consistent with the research results of Xie et al. [49].

As for control variables, GDP of two countries improves China’s RE export in Model (2)-(4). Compared with China’s GDP, GDP of importers has a more significantly positive effect on the export. What can be seen that China’s GDP has no effect on the export, while importers’ is very different in Model (6), which means that China’s RE trade is often affected by the demand for RE minerals. As a part of trade cost, geographical distance will restrain China’s RE export, which is consistent with the expectation of the gravity model. The coefficient estimates of both absolute value of the difference in per capita GDP and economic freedom index are consistent with the above-mentioned conclusions and significant at the level of 1% in Model (6), but they have opposite effects. If there is a large gap in per capita income between the two countries, trade costs will be high, leading export to fall. In Model (4)-(5), the coefficient estimates of absolute value of the difference in per capita GDP is different without controlling economic freedom index, the dummy variable and TFP of the trade partners. In terms of the dummy variable, whether the importer and China disagree on the issue of RE case is not the main reason for RE export to some extent. And, China’s RE export to the United States, the Europe Union and Japan has not declined clearly during the period of RE trade dispute. The regression coefficient estimate of the importer’s TFP, which replaces a country’s technological innovation, is 2.029 and positive at the significance level of 1%, which is in line with the above prediction. It could be interpreted that the better technical innovation of a trade partner is, the stronger demand for special RE minerals in high-tech industries will be, which will lead to the increasing export.

### Product heterogeneity

From the perspective of the RE production chain, the RE production process consists of three steps: extracting RE minerals from mines, processing and smelting metals into useful components and producing end-use applications at the end, which lie in downstream, midstream and upstream, respectively. Considering RE production process, RE products are classified as RE raw materials, RE useful components and RE end-use applications in this paper. Different production chain possesses different environmental pollution level, for metal extraction which adopts the leaching method and reproduction of components which produces fewer pollutants are less harmful to the environment than RE metal processing and smelting. Under the same environmental requirements, the findings analyze whether there is product heterogeneity in the relationship between environmental regulation and China’s RE export. Product classification is listed in Table 1, and the results of product heterogeneity are reported in Table 5.

**Table 5:** Results of product heterogeneity.

Dependent variable	ln*EXP_1g*	ln*EXP_2g*	ln*EXP_3g*
Variable	Model (1)	Model (2)	Model (3)
*ERi*	-0.005	0.094**	-0.027
	(-0.08)	(2.35)	(-1.40)
ln*GDPi*	0.551	-1.287*	0.860**
	(0.56)	(-1.81)	(2.54)
ln*GDPj*	1.081***	1.631***	1.087***
	(7.12)	(10.03)	(15.32)
ln*Dis*	-1.633***	-1.415***	-1.075***
	(-5.99)	(-8.04)	(-13.96)
ln*pgdp*	1.012***	0.054	-0.801***
	(2.82)	(0.22)	(-6.86)
*IEF*	-0.043	0.009	0.049***
	(-1.49)	(0.42)	(4.90)
*TF*	-0.464	-0.253	-0.253
	(-0.77)	(-0.59)	(-1.21)
*TFPj*	-0.877	1.051	1.075**
	(-0.55)	(0.99)	(2.30)
Constant	-23.712	9.608	-25.686***
	(-0.92)	(0.51)	(-2.88)
Estimation method	GLS	GLS	GLS
Observations	340	340	340

Notes: Robust t-statistics are in parentheses

*, **, and *** refer to the significance levels of 10%, 5%, and 1%, respectively.

Model (1)-(3) describe the regression results of the responses of different RE product export to environmental regulation, in which dependent variables are China’s export of RE raw materials (ln*EXP_1g*), RE useful components (ln*EXP_2g*) and RE end-use applications (ln*EXP_3g*). Apparently, the coefficient of environmental regulation is significantly positive in Model (2) and not significant in other models, which demonstrates that environmental regulation plays a vital part in the export of RE useful components in processing and smelting. On the contrary, environmental regulation doesn’t work on the export of other two products statistically, in that more pollutants are produced in the RE processing and smelting process. The coefficient estimate of the core explanatory variable (*ERi*) is 0.094 in Model (2), larger than 0.024 in Model (6) in Table 4. These results imply that RE useful components play a major role in the process by which environmental regulation affects the export in the total sample.

### Mediation test

Through the benchmark regression analysis, it is found that the environmental requirements play a significant role in promoting the export of China’s RE products, especially the RE useful components. Then we are eager to figure out its internal mechanism, trying to explore China’s TFP (*TFPi*) that substitutes for Chinese technological innovation as the mediator variable to examine the mediation test of this paper.

Table 6 shows the results of the mediation test, reporting the test’s three steps. Model (1) is the first step and the estimation of Eq (1) is equal to Model (6) in the benchmark regression (Table 4). Model (2) and Model (3) in Table 6 estimate the results of Eq (2) and Eq (3). We already perceive that environmental regulation positively affects the dependent variable (ln*EXP_g*). Next, the coefficient estimate of the explanatory variable (*ERi*) in Model (2) is significantly positive at the significance level of 1%, indicating domestic environmental regulation enhances Chinese TFP, which runs parallel to some studies [31, 61]. In other words, Chinese technological innovation upgrades with the implementation of environmental regulation. Furthermore, it is found that both environmental regulation and technological innovation positively affect the export in China’s RE industry in Model (3). 0.024, the estimated coefficient of environmental regulation stringency in Model (1), is larger than 0.018 in Model (3), implying that technological innovation has a partial mediation effect. Judging from the regression results by Stata, the proportion of the mediating effect to the total effect is 12.58%.

**Table 6:** Results of the mediation test.

Variable	Model (1)	Model (2)	Model (3)
**Mediator variable**	***TFPi***		
**Dependent variable**	**ln*EXP_g***	***TFPi***	**ln*EXP_g***
*ERi*	0.024**	0.002***	0.018**
	(2.22)	(4.13)	(2.00)
*TFPi*			1.509***
			(9.71)
ln*GDPi*	0.236	0.057***	0.196
	(1.23)	(6.57)	(1.19)
ln*GDPj*	1.096***	0.022***	1.063***
	(73.84)	(56.32)	(75.98)
ln*Dis*	-1.272***	-0.018***	-1.243***
	(-66.62)	(-30.75)	(-60.43)
ln*pgdp*	-0.326***	-0.019***	-0.299***
	(-14.56)	(-27.72)	(-11.33)
*IEF*	0.017***	-0.005***	0.024***
	(8.19)	(-99.53)	(10.76)
*TF*	-0.118	-0.114***	0.017
	(-1.55)	(-16.14)	(0.29)
*TFPj*	2.029***	-0.617***	2.964***
	(29.67)	(-205.54)	(26.54)
Constant	-11.079**	-0.655***	-11.309***
	(-2.20)	(-2.93)	(-2.63)
Estimation method	GLS	GLS	GLS
Observations	340	340	340
**Mediation effect**	**Significant**		

Notes: Robust t-statistics are in parentheses

*, **, and *** refer to the significance levels of 10%, 5%, and 1%, respectively.

### Robustness test

#### The robustness test of benchmark regression

To examine the robustness of benchmark regression, we replace the core explanatory variable (*ERi*) with standard rate of industrial waste water discharge (*ER_wastewater*), comprehensive utilization rate of industrial solid waste (*ER_solidwaste*) and the pollution charge amount (*DF*) [62]. The results of the robustness test are reported in Table 7. We mainly focus on CAC and MBI environmental regulation [33]. Environmental regulation which is calculated by the EWM in the total sample, standard rate of industrial waste water discharge and comprehensive utilization rate of industrial solid waste all belong to CAC environmental regulation. Furthermore, the pollution charge amount is a sort of MBI environmental regulation. Therefore, they could be chosen as alternative variables. The results show that the coefficient estimates of all new explanatory variables (*ER_solidwaste*, *ER_wastewater and DF*) are positive and significant, verifying the prediction results and further ensuring the robustness of the basic model.

**Table 7:** The robustness test of benchmark regression.

Variable	Model (1)	Model (2)	Model (3)
*ER_wastewater*	0.049***		
	(26.88)		
*ER_solidwaste*		0.113***	
		(19.49)	
*DF*			0.006**
			(2.46)
ln*GDPi*	0.346***	-0.359***	0.230
	(10.70)	(-5.72)	(1.27)
ln*GDPj*	1.082***	1.086***	1.094***
	(70.78)	(75.02)	(75.03)
ln*Dis*	-1.253***	-1.271***	-1.276***
	(-72.80)	(-70.34)	(-67.02)
ln*pgdp*	-0.299***	-0.334***	-0.344***
	(-15.20)	(-16.36)	(-15.55)
*IEF*	0.015***	0.015***	0.018***
	(8.81)	(8.39)	(8.74)
*TF*	0.642***	0.392***	-0.080
	(9.49)	(7.23)	(-1.07)
*TFPj*	1.700***	1.942***	2.068***
	(27.34)	(29.70)	(30.18)
Constant	-16.645***	1.310	-10.297**
	(-17.21)	(0.83)	(-2.09)
Estimation method	GLS	GLS	GLS
Observations	340	340	340

Notes: Robust t-statistics are in parentheses

*, **, and *** refer to the significance levels of 10%, 5%, and 1%, respectively.

#### The robustness test of product heterogeneity

In this section, we will alter the dependent variable to test whether the environmental regulation is indeed related to RE export in sub-samples, by selecting the export of representative products related to useful components and end-use applications for regression. The robustness results of product heterogeneity analysis are seen in Table 8, with the first two models belonging to RE useful components and the latter models describing RE end-use applications.

**Table 8:** The robustness test of product heterogeneity.

HS code	284610	284690	720299	850511
Variable	Model (1)	Model (2)	Model (3)	Model (4)
*ERi*	0.098***	0.087***	-0.015	-0.006
	(4.16)	(3.88)	(-0.99)	(-0.40)
ln*GDPi*	-3.251***	-2.706***	0.528*	0.553**
	(-7.61)	(-6.62)	(1.93)	(2.01)
ln*GDPj*	1.212***	1.954***	0.772***	1.303***
	(15.71)	(21.19)	(13.92)	(25.87)
ln*Dis*	-1.209***	-1.447***	-1.076***	-1.118***
	(-10.22)	(-11.06)	(-19.09)	(-15.66)
ln*pgdp*	0.829***	-0.051	-0.534***	-0.444***
	(4.13)	(-0.26)	(-6.08)	(-4.79)
*IEF*	-0.060***	-0.006	0.038***	0.009
	(-4.44)	(-0.39)	(4.36)	(1.10)
*TF*	0.842***	0.804***	-0.354*	-0.438***
	(3.19)	(3.14)	(-1.95)	(-2.62)
*TFPj*	1.670*	3.206***	1.109***	1.074***
	(1.93)	(3.61)	(3.03)	(3.18)
Constant	71.841***	41.411***	-10.155	-25.564***
	(6.49)	(3.92)	(-1.41)	(-3.55)
Estimation method	GLS	GLS	GLS	GLS
Observations	233	243	326	326

Notes: Robust t-statistics are in parentheses

*, **, and *** refer to the significance levels of 10%, 5%, and 1%, respectively.

Four products are chosen in Table 8, including cerium compounds, compounds of rare earth metals excluding cerium, ferro alloys and magnets. Besides, the four products have different HS codes. Cerium compounds have 284610, compounds of rare earth metals excluding cerium have 284690, ferro alloys have 720299 and magnets have 850511. As illustrated in Table 8, the regression results of RE useful applications are parallel to Model (2) in Table 5, positively and significantly at the level of 1%. Apart from this, the sign and value of the core explanatory variable’s coefficient in Model (3)-(4) keep consistent with the results of RE end-use applications in Table 5. These can explain the robustness of empirical results.

#### The robustness test of mediation test

Some researchers hold the view that the impact of different kinds of environmental regulations on TFP and industrial upgrading can cause heterogeneity [63, 64]. In order to examine whether the mediator variable (*TFPi*) is stable in the effect mechanism of environmental regulation on RE export, standard rate of industrial waste water discharge (*ER_wastewater*) and comprehensive utilization rate of industrial solid waste (*ER_solidwaste*) are used as the alternative variables of the explanatory variable. The results of the robustness of mediation test are shown in Table 9.

**Table 9:** The robustness test of mediation test.

Variable	Model (1)	Model (2)	Model (3)	Model (4)	Model (5)	Model (6)
Mediator variable	*TFPi*					
Dependent variable	ln*EXP_g*	TFPi	ln*EXP_g*	ln*EXP_g*	TFPi	ln*EXP_g*
*ER_wastewater*	0.049***	0.001***	0.046***			
	(26.88)	(8.99)	(21.20)			
*ER_solidwaste*				0.113***	0.004***	0.106***
				(19.49)	(14.33)	(16.75)
*TFPi*			0.788***			0.697***
			(6.53)			(6.02)
ln*GDPi*	0.346***	0.084***	0.283***	-0.359***	0.051***	-0.362***
	(10.70)	(42.61)	(7.72)	(-5.72)	(15.36)	(-5.30)
ln*GDPj*	1.082***	0.022***	1.065***	1.086***	0.022***	1.072***
	(70.78)	(54.98)	(69.62)	(75.02)	(54.08)	(75.16)
ln*Dis*	-1.253***	-0.017***	-1.231***	-1.271***	-0.018***	-1.252***
	(-72.80)	(-30.09)	(-68.44)	(-70.34)	(-30.26)	(-66.79)
ln*pgdp*	-0.299***	-0.019***	-0.303***	-0.334***	-0.019***	-0.333***
	(-15.20)	(-27.54)	(-12.55)	(-16.36)	(-28.32)	(-13.58)
*IEF*	0.015***	-0.005***	0.020***	0.015***	-0.005***	0.020***
	(8.81)	(-101.34)	(10.03)	(8.39)	(-99.17)	(9.63)
*TF*	0.642***	-0.103***	0.656***	0.392***	-0.094***	0.420***
	(9.49)	(-15.80)	(7.65)	(7.23)	(-15.35)	(6.87)
*TFPj*	1.700***	-0.621***	2.204***	1.942***	-0.620***	2.388***
	(27.34)	(-208.58)	(23.30)	(29.70)	(-208.71)	(25.55)
Constant	-16.645***	-1.411***	-15.481***	1.310	-0.615***	1.006
	(-17.21)	(-25.03)	(-14.60)	(0.83)	(-7.37)	(0.59)
Estimation method	GLS	GLS	GLS	GLS	GLS	GLS
Conservations	340	340	340	340	340	340
**Mediation effect**	**Significant**			**Significant**		

Notes: Robust t-statistics are in parentheses

*, **, and *** refer to the significance levels of 10%, 5%, and 1%, respectively.

Model (1)-(3) represent the mediation effect of standard rate of industrial waste water discharge, with a positive coefficient of the explanatory variable at the significance level of 1%. The results of comprehensive utilization rate of industrial solid waste are reported in Model (4)-(6) and show that technological innovation indeed has a partial mediation effect, which can verify the robustness of the mediation test.

## Discussion

This paper uses China’s RE product export data to 17 countries and an extended gravity model to investigate the impact of environmental regulation on RE export. The findings suggest that environmental regulation can significantly promote RE export, which are parallel to the research results of Porter and van der Linde [19], Berman and Bin [20] and Cantore and Cheng [21]. They all believe that the “innovation offsets” effect of strict environmental regulation is conducive to enhancing China’s export competitiveness. And, it also proves the existence of the strong PH [22].

In addition, the analysis of product heterogeneity reveals that environmental regulation has a different effect on different RE products. For RE useful components produced in the process of RE processing and smelting, environmental regulation tends to play a dominant and positive role in the export. However, for RE raw materials and RE end-use applications, environmental regulation inhibits the export, but the results are not significant. A possible reason for this is that RE processing and smelting can produce more pollutants than RE raw materials and RE end-use applications. Therefore, in the mechanism, there exists the product heterogeneity under the same circumstances.

What’s more, the results of the mediation test to some extent supplement the existing research, and prove that the weak PH is true [23, 24, 32]. Further calculation reports that the mediation effect of technological innovation is 0.1258. This study suggests that the main effect of environmental regulation on China’s manufacturing industry is “innovation offsets”, which is beneficial to China’s overall export of manufacturing. It also supports the above benchmark regression results.

## Conclusion and policy recommendations

### Conclusion

This study investigates the relationship between environmental regulation and the export from the perspective of product rather than country or industry. And, RE products are divided into RE raw materials, RE useful components and RE end-use applications to find out whether there is product heterogeneity in the mechanism. Furthermore, we empirically examine its possible mediation effect. The following conclusions come from the above analysis. Firstly, environmental regulation has a significantly positive influence on China’s RE export. This shows that RE export trade is vulnerable to the demand chain. If China has a large economy, it tends to develop technology aggressively to increase export. Secondly, product heterogeneity exists in the process of environmental regulation affecting the export. For RE useful components, environmental regulation takes a lead to promote the export. That is, in the process of RE processing and smelting, the produced pollutants are much more than that in RE mining or final production. In contrast, environmental regulation inhibits but with no significant effect on RE raw material and RE end-use application export. Finally, technological innovation is a possible channel through which environmental regulation affects the export. In particular, strict environmental regulation has led to technological innovation, improved productivity and increased export.

### Policy recommendations

Furthermore, this paper also puts forward the following important policy recommendations for China’s environmental protection. First, we should strengthen environmental regulation and perfect the environmental regulation system. China should implement better environmental and labor standards [65], for instance the United States puts strict rules on the treatment of RE ore tailings. Second, pollutants from RE mining are supposed to be paid more attention. It is found that environmental requirements have a positive effect on RE useful component export. However, this study shows that environmental regulation has no obvious effect on RE raw material export. Taxes on RE resource mining and environmental protection should be raised to protect environment. Third, the Chinese government needs to support enterprises to improve their technological innovation capability. Finally, we should use international trade rules to resolve trade disputes.

However, there are also limitations in this paper. Our findings are based on the limited availability of data. Moreover, the impact of environmental regulation on the export is a complex process, and there may be multiple mechanisms. In view of this, this paper should be considered as a preliminary analysis, and more detailed and in-depth research is still needed.

## Supporting information

S1 Table The data of all variables. (XLSX)
